# Phosphodiesterase Type 5 Inhibitors Synergize Vincristine in Killing Castration-Resistant Prostate Cancer Through Amplifying Mitotic Arrest Signaling

**DOI:** 10.3389/fonc.2020.01274

**Published:** 2020-08-07

**Authors:** Jui-Ling Hsu, Wohn-Jenn Leu, Lih-Ching Hsu, Chen-Hsun Ho, Shih-Ping Liu, Jih-Hwa Guh

**Affiliations:** ^1^School of Pharmacy, College of Medicine, National Taiwan University, Taipei, Taiwan; ^2^Department of Pharmacy, New Taipei Municipal TuCheng Hospital, Chang Gung Memorial Hospital, New Taipei city, Taiwan; ^3^Department of Urology, Shuang Ho Hospital, Taipei Medical University, Taipei, Taiwan; ^4^Department of Urology, School of Medicine, College of Medicine, Taipei Medical University, Taipei, Taiwan; ^5^Department of Urology, National Taiwan University Hospital College of Medicine, Taipei, Taiwan

**Keywords:** sildenafil, vincristine, castration-resistant prostate cancer, spindle assembly checkpoint, kinetochore tension

## Abstract

Combination therapies that display cancer-killing activities through either coexistent targeting of several cellular factors or more efficient suppression of a specific pathway are generally used in cancer treatment. Sildenafil, a specific phosphodiesterase type 5 (PDE5) inhibitor, has been suggested to display both cardioprotective and neuroprotective activities that provide a rationale for the combination with vincristine on the treatment against castration-resistant prostate cancer (CRPC). In the present work, vincristine arrested cells in the metaphase stage of mitosis. Vincristine-induced mitotic arrest was identified by Cdk1 activation (i.e., increased Cdk1^Thr161^ phosphorylation and decreased Cdk1^Tyr15^ phosphorylation), cyclin B1 upregulation, and increased phosphorylation of multiple mitotic proteins and stathmin. Sildenafil synergistically potentiated vincristine-induced mitotic arrest and a dramatic increase of mitotic index. Furthermore, sildenafil potentiated vincristine-induced mitochondrial damage, including Mcl-1 downregulation, Bcl-2 phosphorylation and downregulation, Bak upregulation and loss of mitochondrial membrane potential, and sensitized caspase-dependent apoptotic cell death. Sildenafil-mediated synergistic effects were mimicked by other PDE5 inhibitors including vardenafil and tadalafil, and also by *PDE5A* knockdown in cells, suggesting PDE5-involved mechanism. Notably, sildenafil amplified vincristine-induced phosphorylation and cleavage of BUBR1, a protein kinase in spindle assembly checkpoint (SAC) function and chromosome segregation. Sildenafil also significantly decreased kinetochore tension during SAC activation. Moreover, sildenafil synergized with vincristine on suppressing tumor growth in an *in vivo* model. In conclusion, the data suggest that sildenafil, in a PDE5-dependent manner, potentiates vincristine-induced mitotic arrest signaling, and sensitizes mitochondria damage–involved apoptosis in CRPC. Both *in vitro* and *in vivo* data suggest the combination potential of PDE5 inhibitors and vincristine on CRPC treatment.

## Introduction

Prostate cancer is the second most commonly occurring cancer in men worldwide. Prostate cancer that keeps growing regardless of androgen-deprivation therapy in the situation of very low serum testosterone levels is considered castration-resistant prostate cancer (CRPC). New therapies have emerged for treating CRPC because of better understanding of the molecular signaling pathways underlying the progression and development of CRPC (1, 2). However, even though numerous treatment options have been provided, the patients only have limited survival benefit (3, 4). Recently, several therapeutic agents have been introduced to treat CRPC to improve overall survival; the clinicians still face the critical challenge in choice of the best treatment sequencing (2). In fact, the therapy is still in evolution and new clinical insights need to be proposed. *Vinca* alkaloids (e.g., vincristine, vinblastine, vinorelbine, and vindesine) are a family of anti-mitotic and anti-microtubule agents widely used in cancer chemotherapy. The combination of *Vinca* alkaloids with several anticancer drugs in CRPC treatment has been demonstrated to display favorable activity and a low toxicity profile in several clinical studies (5–7). These combination therapies fulfill the purpose of mechanism-based killing cancer and reduction of toxic effect through decreased doses of individual drugs and suggest that *Vinca* alkaloids are options in combination with other therapeutic drugs in CRPC treatment.

Sildenafil, which acts by inhibiting phosphodiesterase type 5 (PDE5), is a medication for the treatment of erectile dysfunction and pulmonary arterial hypertension (8, 9). Recent evidence has demonstrated the cardioprotective activity of sildenafil against myocardial injury by ischemia/reperfusion, heart failure, cardiac hypertrophy, and diabetic cardiomyopathy (10, 11). Furthermore, a variety of studies have revealed the neuroprotective role of sildenafil and have suggested that sildenafil could be repurposed as a potential therapeutic drug for the treatment of several neuronal disorders (12, 13). Moreover, the anti-inflammatory effects of sildenafil have been proposed to show therapeutic benefit in cardiac and inflammatory complications (10). Notably, sildenafil has been reported to induce apoptotic sensitization of several types of cancer to chemotherapeutic drugs, including prostate cancer, breast cancer, and small cell and non-small cell lung cancers (10, 14–16). It has been suggested that co-treatment of sildenafil and vincristine increases apoptotic sensitization of halaven-resistant KBV20C cancer cells (17). Combination of sildenafil with standard chemotherapy agents (vincristine/etoposide/cisplatin) significantly enhances anticancer effect against medulloblastoma (18). These studies suggest the feasibility and therapeutic anticancer potential between the combination of sildenafil with vincristine. There is an ongoing interest by both basic and clinical oncologic investigators in discovering their clinical uses. In the present work, the anticancer sensitization of sildenafil on vincristine-treated CRPC has been studied. To the best of our knowledge, this is the first study dealing with the underlying mechanism related to perturbation of spindle checkpoint protein and microtubule–kinetochore interactions in sildenafil-sensitized anticancer effect.

## Materials and Methods

### Materials

Human prostate adenocarcinoma cell lines, PC-3 and DU-145, were obtained from American Type Culture Collection (Rockville, MD, USA). RPMI 1640 medium, fetal bovine serum (FBS), penicillin, and streptomycin were purchased from GIBCO/BRL Life Technologies (Grand Island, NY). Antibodies of PARP-1, Bcl-2, Bcl-xL, Bak, Mcl-1, α-tubulin, cyclin A, cyclin B, cyclin-dependent kinase (Cdk) 1, and GAPDH were obtained from Santa Cruz Biotechnology (Santa Cruz, CA). Antibodies of cleaved caspase-9, caspase-8, β-tubulin (Alexa Fluor 594 Conjugate), p-Cdk1^Thr161^, and p-Cdk1^Tyr15^ were from Cell Signaling Technologies (Boston, MA). Stathmin-1, BUBR1, and CENP-A were from Abcam (Cambridge, UK). MPM2 was from Millipore (Bedford, MA, USA). Caspase-3 was purchased from Imgenex (San Diego, CA). Antibody of PDE5 was from OriGene Technologies (Rockville, MD, USA). PDE5 small interfering RNA (siRNA) was from GE Healthcare Dharmacon (Chicago, USA). JC-1 and DAPI were from Molecular Probes (Eugene, OR, USA). Anti-mouse and anti-rabbit IgGs were from Jackson ImmunoResearch Laboratories (West Grove, PA, USA). Leupeptin, phosphatase inhibitors (NaF and Na_3_VO_4_), dithiothreitol, phenylmethylsulfonylfluoride (PMSF), propidium iodide (PI), and all other chemical compounds were purchased from Sigma-Aldrich (St. Louis, MO, USA).

### Cell Culture

PC-3 and DU145 cells were cultured in RPMI 1640 medium supplemented with 5% FBS (*v*/*v*), penicillin (100 U/ml), and streptomycin (100 μg/ml). Cultures were maintained in a 37°C incubator with 5% CO_2_. Adherent cultures were passaged using 0.05% trypsin–EDTA after reaching 80% confluence.

### Flow Cytometric Assay With PI Staining

Cells were harvested by trypsinization, fixed with 70% (*v*/*v*) alcohol at 4°C for 30 min and washed with phosphate-buffered saline (PBS). After centrifugation, cells were centrifuged and re-suspended with 0.3 ml PI solution containing Triton X-100 (0.1% *v*/*v*), RNase (100 μg/ml), and PI (80 μg/ml). DNA content was analyzed with the FACScan and CellQuest software (Becton Dickinson, Mountain View, CA).

### DNA Fragmentation Assay

DNA fragmentation was determined using commercial Cell Death Detection ELISA^PLUS^ kit (Roche, Mannheim, Germany) which was based on the examination of cytoplasmic histone-associated DNA fragments (mono- and oligo-nucleosomes) in cells after the induction of cell apoptosis. After the indicated treatment, the cells were lysed and centrifuged, and the supernatant was used for the detection of nucleosomal DNA fragments according to the manufacturer's protocol.

### Cell-Cycle Synchronization

Synchronization of the cells was performed by double thymidine block. Briefly, the cells were treated with 2 mM thymidine in medium/10% FBS for 12 h. After washing cells with PBS, the block was released by the incubation of cells in the medium without thymidine and then followed by another 12-h thymidine block. The cells were harvested at the indicated times. The cell-cycle progression was detected by flow cytometric analysis and analyzed with CellQuest software (Becton Dickinson).

### Western Blotting

After the treatment, cells were harvested with trypsinization, centrifuged, and lysed in 50 μl of lysis buffer containing 10 mM Tris–HCl (pH 7.4), 150 mM NaCl, 1 mM EGTA, 1% Triton X-100, 1 mM PMSF, 10 μg/ml leupeptin, 1 mM dithiothreitol, 1 mM NaF, and 1 mM sodium orthovanadate. Total protein was quantified, mixed with sample buffer, and boiled at 90°C for 5 min. An equal amount of protein (30 μg) was separated by electrophoresis in 8 or 12% SDS-PAGE, transferred to PVDF membranes. After 1-h incubation at room temperature in PBS/0.1% Tween 20/5% non-fat milk, the membrane was washed with PBS/0.1% Tween 20 for 1 h and immuno-reacted with the indicated antibody for 1 h at room temperature. After three washings with PBS/0.1% Tween 20, the anti-mouse or anti-rabbit IgG (dilute 1:8000) was applied to the membranes for 1 h at room temperature. The membranes were washed with PBS/0.1% Tween 20 for 1 h and the detection of signal was performed with an enhanced chemiluminescence detection kit (Amersham, Buckinghamshire, UK).

### Measurement of Mitochondrial Membrane Potential (ΔΨ_m_)

JC-1, a mitochondrial dye staining mitochondria in living cells in a membrane potential–dependent fashion, was used to determine ΔΨ_m_. Cells were treated with or without the compound. Thirty minutes before termination of incubation, cells were incubated with JC-1 (5 μM) at 37°C for 10 min. Accumulation of JC-1 was determined using flow cytometry analysis (Becton Dickinson, Mountain View, CA).

### siRNA Transfection

Cells were seeded into a six-well-plate with 30% confluence and grown for 24 h to 50% confluence. Each well was washed twice with PBS and 1 ml of serum-free Opti-MEM (Life Technologies, Ground Island, NY) was added. Aliquots containing control or PDE5 siRNA (a pooled siRNA sequence other than a single sequence) in serum-free Opti-MEM were transfected into cells using Lipofectamine 2000 according to the manufacturer's instructions. After transfection for 6 h, cells were washed twice with PBS and incubated in 10% FBS-containing RPMI-1640 medium for 48 h, and the subsequent experiments were performed.

### Confocal Immunofluorescence Microscopic Examination

For β-tubulin and CENP-A staining, cells were fixed with 100% methanol (−20°C) for 5 min and incubated in 1% bovine serum albumin (BSA)/PBS containing 0.1% Triton X-100 at 37°C for 30 min. Cells were washed and stained with β-tubulin antibody at 37°C for 1 h or stained with CENP-A antibody at 4°C overnight. Cells were next incubated with FITC-conjugated secondary antibody at room temperature for 1 h. For BUBR1 staining, cells were fixed with 4% paraformaldehyde in PBS for 20 min, permeabilized with 0.1% Triton X-100 for 10 min, and blocked with 5% BSA/PBS for 1 h. Cells were washed and stained with BUBR1 antibody at 4°C and then FITC-conjugated secondary antibody at room temperature for 1 h. Nuclear identification was performed by DAPI staining. The air-dried coverslips were next mounted onto glass slides using ProLongR Diamond Antifade Mountant (Thermo Fisher Scientific, Waltham, MA, USA). The cells were analyzed by a confocal microscope Zeiss LSM88 (Carl Zeiss, Jena, Germany). As for the measurement of a mitotic index, the number of cells in mitosis (prophase, metaphase, anaphase, and telophase) was divided by the total number of cells. As for the measurement of sister kinetochore distance, distances between paired kinetochores (*n* = 50) were measured at individual z planes using the ZEN 2012 (black edition) software (19).

### *In vivo* Anti-tumor Study

PC-3-derived cancer xenografts in nude mice were used as an *in vivo* model. The nude mice were subcutaneously injected with PC-3 cells (10^7^ cells/mouse). When the tumor volume reached 400–600 mm^3^, the mice were divided into four groups (*n* = 7–9) and compound treatment was initiated. The animals received intraperitoneal injections of 5% DMSO (for control), vincristine alone (0.5 mg/kg, once weekly), sildenafil alone (10 mg/kg, 5 on 2 off), or vincristine plus sildenafil. The tumor length (*l*) and width (*w*) were measured to obtain tumor volume as *lw*^2^/2. The protocols of the *in vivo* study were approved by the Animal Care and Use Committee at National Taiwan University. All animal procedures and protocols were approved by an AAALAC-accredited facility.

### Data Analysis

Data are presented as mean ± SEM. Statistical analysis was performed and two-group comparisons were done with Student's *t*-test. *P* < 0.05 was considered statistically significant.

## Results

### Sildenafil Sensitizes Vincristine-Induced Cell Death and an Increase of Mitotic Index

**Figure 1:**
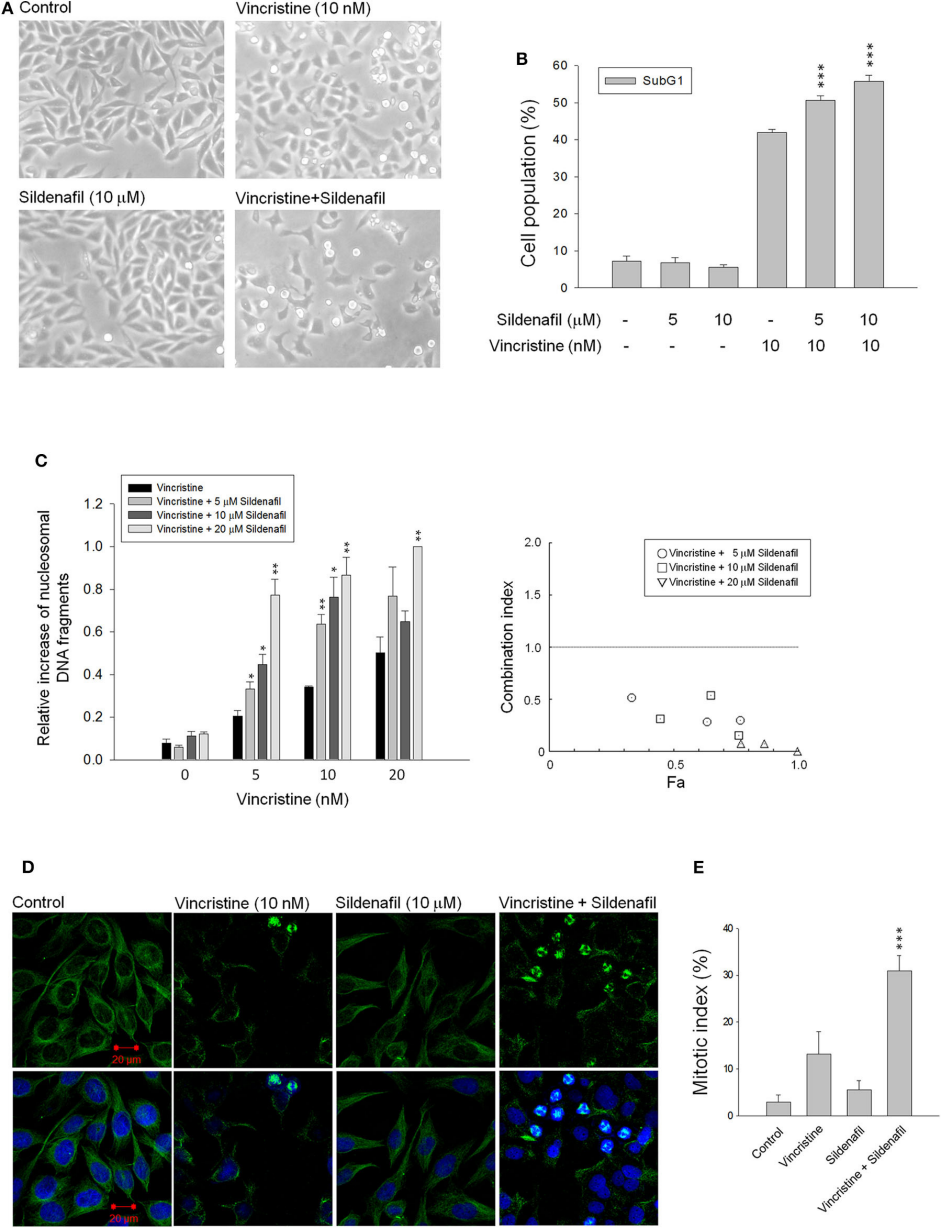
Effect of vincristine and sildenafil on cell morphology, apoptosis, and mitotic index in PC-3 cells. The cells were incubated in the absence or presence of the indicated agent for 24 h **(A,C–E)** or 48 h **(B)**. The cell morphology was observed under microscopic examination **(A)**, or the cells were harvested for propidium iodide staining to analyze the distribution of cell populations at sub-G1 (apoptosis) phase using FACScan flow cytometric analysis **(B)**, or the cell apoptosis was examined through measuring the level of nucleosomal DNA fragments **(C)**. The confocal immunofluorescence examination was performed to detect microtubule (green) and chromosome (blue) using β-tubulin antibody and DAPI **(D)**, and the quantitative mitotic index was obtained accordingly **(E)**. Data are expressed as mean ± SEM of three to nine independent determinations. ^*^P < 0.05, ^**^P < 0.01, and ^***^P < 0.001 compared with vincristine alone.

Vincristine is a natural alkaloid working predominantly by binding to tubulin proteins, preventing their polymerization and microtubule formation, leading to failure of chromosome separation during the metaphase and eventually causing cell apoptosis. The cell morphology analysis via microscopic examination in Figure 1A shows that vincristine induced morphological change and cell shrinkage, a hallmark of apoptotic mode of programmed cell death, of PC-3 cells. Sildenafil profoundly exacerbated vincristine-induced effect. The data were substantiated using flow cytometric quantitation of DNA content showing that sildenafil synergistically increased vincristine-induced apoptotic sub-G1 cell population in PC-3 cells (Figure 1B). Sildenafil-induced apoptotic potentiation was substantiated by the detection of nucleosomal DNA fragments. The synergism between vincristine and sildenafil was assessed through constructing isobolograms and calculating combination index (CI) values using Chou–Talalay method (20). The resulting CI values were <1.0 confirming the synergistic effects. Similar data of synergistic apoptotic effect also were obtained in DU-145 cells (Supplementary Figure 1). Furthermore, the mitotic abnormalities, key characteristics of anti-mitotic agents, were detected using double staining of mitotic spindle and chromosome. The images depicted in Figure 1C show that vincristine induced mitotic arrest with abnormal features of mitosis. The effect was dramatically exacerbated in the presence of sildenafil. To further substantiate the effect on mitotic arrest, the cells were synchronized using double thymidine block to arrest cell at G1/S boundary. After the release from double thymidine block, cell-cycle progression, and cell population at distinct phase were detected at different time points (Supplementary Figure 2). The data showed that in the presence of vincristine at 17, 21, and 24-h treatment, about 23–31% of the cell population was capable of entering G1 phase. In contrast, the population was significantly reduced in the combinatory treatment of vincristine and sildenafil; furthermore, sildenafil significantly increased vincristine-induced G2/M population (Supplementary Figure 2).

The levels of mitotic arrest in cells responsive to microtubule-targeting agents are proportional to those of subsequent cell death. Accordingly, the mitotic index was measured showing that sildenafil significantly increased vincristine-induced mitotic arrest (Figure 1D) and mitotic index (Figure 1E). The mitotic index was defined in detail in characteristics of several mitotic phases, including prophase, metaphase, anaphase, and telophase. The data in Table 1 demonstrated that vincristine caused predominantly an increase of cell population in metaphase, such as unaligned chromosome, tripolar spindle, multiple spindle poles, and asymmetrical bipolar spindle. The presence of sildenafil dramatically increased the probability of cells at metaphase, in particular tripolar spindle and multiple spindle poles, in cells (Table 1). Besides, it has been evident that cells can survive metaphase arrest at a sublethal concentration of vincristine possibly through completing cytokinesis normally (21). Our data showed that sildenafil decreased, although not significantly, the level of cytokinesis in cells responsive to vincristine (Table 1).

**Table 1:** Effect of vincristine alone and combined with sildenafil on several mitotic phases in PC-3 cells.

**Cell phase**	**Feature**	**Control**	**Vincristine**	**Sildenafil**	**Vincristine + Sildenafil**
Prophase	Normal	0.79 ± 0.06	1.23 ± 0.42	1.08 ± 0.23	1.05 ± 0.16
	Monopolar	0	1.02 ± 0.83	0	0.26 ± 0.26
Metaphase	Normal	1.47 ± 0.73	0.58 ± 0.085	1.05 ± 0.53	0.34 ± 0.20
	Unaligned chromosome	0	2.44 ± 0.21	0.23 ± 0.19	1.30 ± 0.39
	Tripolar	0	2.00 ± 1.28	0	7.98 ± 0.45^a^
	Multiple spindle poles	0	4.30 ± 0.72	0	18.40 ± 1.02^b^
	Asymmetrical bipolar spindle	0	0.50 ± 0.32	0	0.11 ± 0.11
Anaphase	Normal	0.25 ± 0.25	0.18 ± 0.18	0	0.11 ± 0.11
	Lagging chromosomes	0	0.22 ± 0.22	0	0.24 ± 0.12
Telophase	Normal	0.48 ± 0.48	0.23 ± 0.23	3.23 ± 0.99	0.11 ± 0.11
Cytokinesis		2.02 ± 0.86	0.45 ± 0.45	5.58 ± 1.27	0.13 ± 0.13

a *P < 0.05 compared with vincristine alone*.

b *P < 0.001 compared with vincristine alone*.

### Sildenafil Exacerbates Vincristine-Induced Mitotic Arrest Signaling and Mitochondrial Damage Response

**Figure 2:**
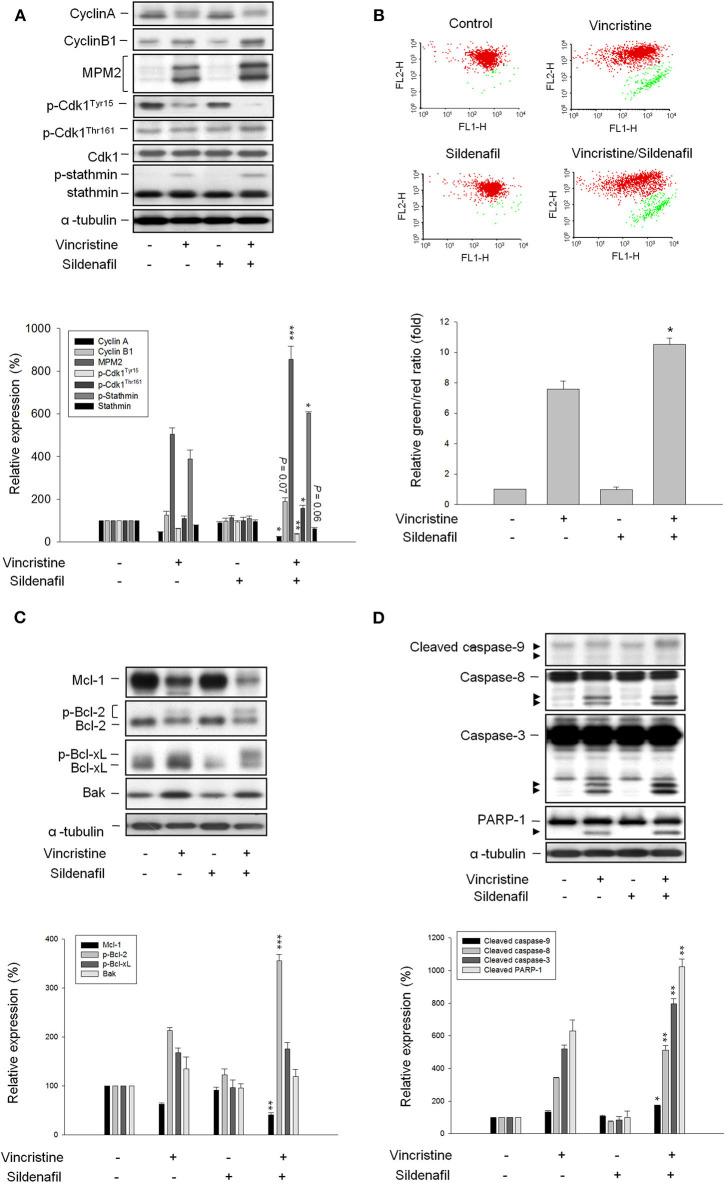
Effect of vincristine and sildenafil on cell-cycle regulators and mitochondria-involved signaling pathway. PC-3 cells were treated in the absence or presence of vincristine (10 nM) and sildenafil (10 μM) for 24 h **(A,C,D)** or 48 h **(B)**. After the treatment, the cells were harvested and lysed for the detection of protein expressions of cell-cycle regulators **(A,C,D)** by Western blot analysis or the cells were harvested for JC-1 staining to detect mitochondrial membrane potential using FACScan flow cytometric analysis **(B)**. Data are expressed as mean ± SEM of three to six determinations. ^*^P < 0.05, ^**^P < 0.01, and ^***^P < 0.001 compared with vincristine alone.

It has been widely recognized that exposure of cells to anti-tubulin agents always leads to prolonged activation of spindle assembly checkpoint (SAC), resulting in mitotic arrest and eventually cell apoptosis (22, 23). Cdk1 activation needs a multiple process including Cdk1/cyclin B1 complex formation and nuclear relocation, and is based on phosphorylation/dephosphorylation. Dephosphorylation of both Thr14 and Tyr15 is necessary for kinase activity. On the contrary, Thr161 must be phosphorylated for activity (24, 25). As expected, vincristine induced the upregulation of cyclin B1 protein expression associated with a decrease of cyclin A protein levels, and caused an increase of Cdk1^Thr161^ phosphorylation and a decrease of Cdk1^Tyr15^ phosphorylation suggesting the induction of mitotic arrest (Figure 2A). Increased phosphorylation of multiple mitotic proteins (MPM-2) and stathmin, which regulate the dynamics of microtubule polymerization and depolymerization, further validate the mitotic arrest to vincristine action. Notably, vincristine-mediated signaling in mitotic arrest was significantly amplified in the presence of sildenafil (Figure 2A).

Several lines of evidence suggest a link between the network of SAC and mitochondrial functions that may regulate cellular signaling to cell death (26). Accordingly, JC-1 mitochondrial membrane potential assay was performed and the data demonstrated that vincristine induced a loss of mitochondrial membrane potential that was significantly exacerbated in the presence of sildenafil, suggesting further mitochondrial damage to sildenafil action (Figure 2B). Mitochondrial outer membrane potential permeabilization, which is controlled by Bcl-2 family members, is a key event in apoptotic insult because it induces the release of proapoptotic proteins to the cytosol. Vincristine induced downregulation of Mcl-1 and Bcl-2 (two anti-apoptotic Bcl-2 family members) and upregulation of Bak (a pro-apoptotic member) that explained the mitochondrial damage (Figure 2C). Moreover, Bcl-2 phosphorylation was evoked (Figure 2C) that further verifies the mitotic arrest because it has been evident that Cdk1/cyclin B1-mediated Bcl-2 phosphorylation serves as a functional link coupling mitotic arrest and cell death (27). Of note, sildenafil profoundly aggravated vincristine-mediated effects, in particular the Bcl-2 phosphorylation (Figure 2C). The data together with the amplification of caspase activation including caspase-8, -9, and -3, and increased cleavage of PARP-1 (a caspase-3 substrate) (Figure 2D) confirmed the synergistic effect on mitotic arrest and apoptotic cell death.

### Other PDE5 Inhibitors Mimic Sildenafil on Potentiating Vincristine-Induced Effects

**Figure 3:**
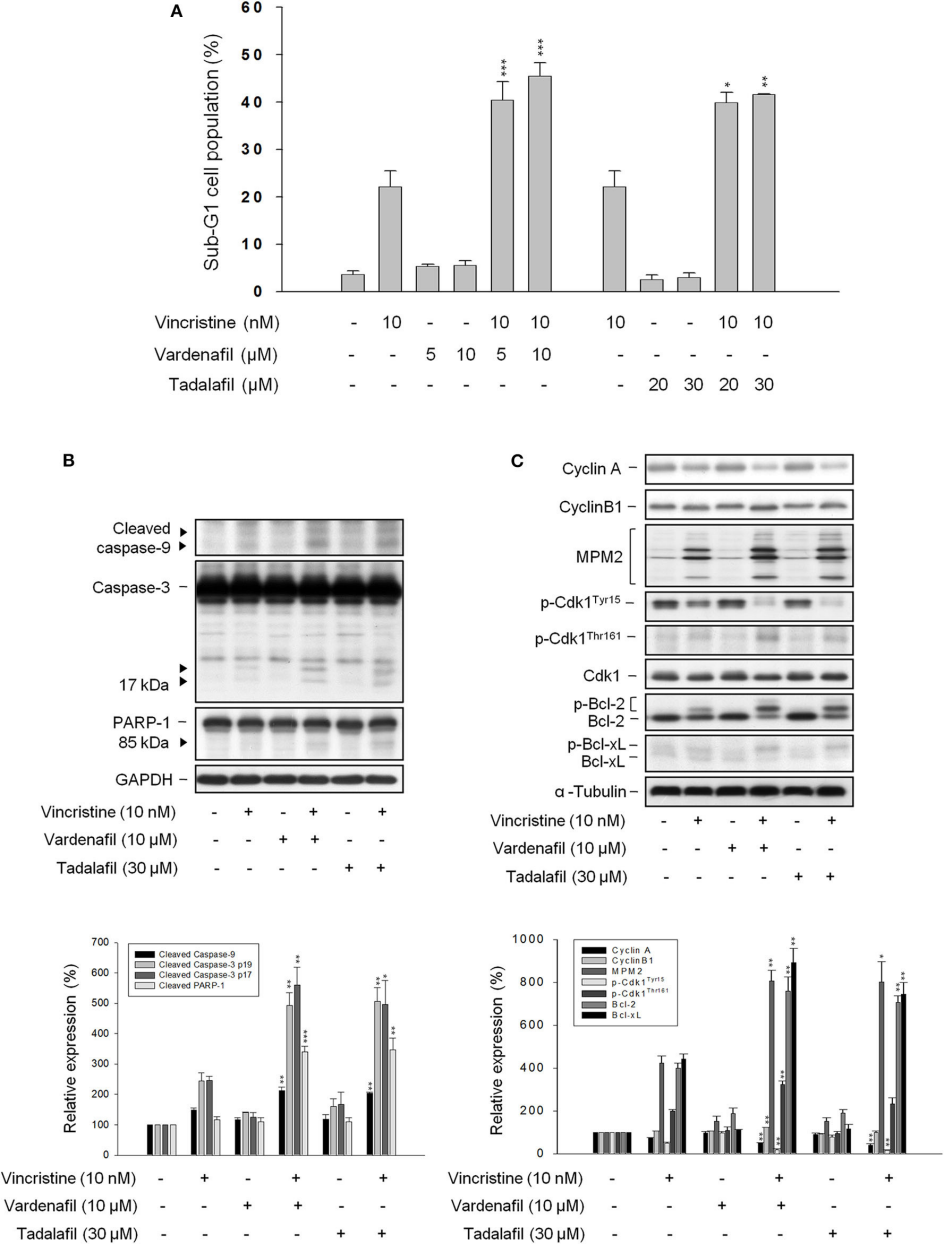
Effect of vardenafil and tadalafil on vincristine-induced sub-G1 population and protein expression. PC-3 cells were incubated in the absence or presence of the indicated agent for 48 h **(A)** or 24 h **(B,C)**. The cells were harvested for propidium iodide staining to analyze the distribution of cell populations at sub-G1 (apoptosis) phase using FACScan flow cytometric analysis **(A)**, or the cells were harvested and lysed for the detection of protein expression by Western blot analysis **(B,C)**. Data are expressed as mean ± SEM of three independent determinations. ^*^P < 0.05, ^**^P < 0.01, and ^***^P < 0.001 compared with vincristine alone.

The effect of other PDE5 inhibitors including vardenafil and tadalafil on vincristine-induced cell apoptosis and related signaling pathway was examined. Both vardenafil and tadalafil sensitized apoptotic cell death to vincristine action in PC-3 cells (Figure 3A) and DU-145 cells (Supplementary Figure 3); the activation of caspase cascade also was potentiated (Figure 3B). Both vardenafil and tadalafil synergistically exaggerated vincristine-induced signaling pathways on mitotic arrest effects, including downregulation of cyclin A whereas upregulation of cyclin B1 protein expression, increased mitotic-specific MPM-2 phosphorylation, increased Cdk1 activity (i.e., decreased Cdk1^Tyr15^ phosphorylation associated with increased Cdk1^Thr161^ phosphorylation), and increased phosphorylation of Bcl-2 and Bcl-xL (Figure 3C).

### Vincristine-Induced Effects Are Amplified in Cells With PDE5A Gene Knockdown by siRNA

**Figure 4:**
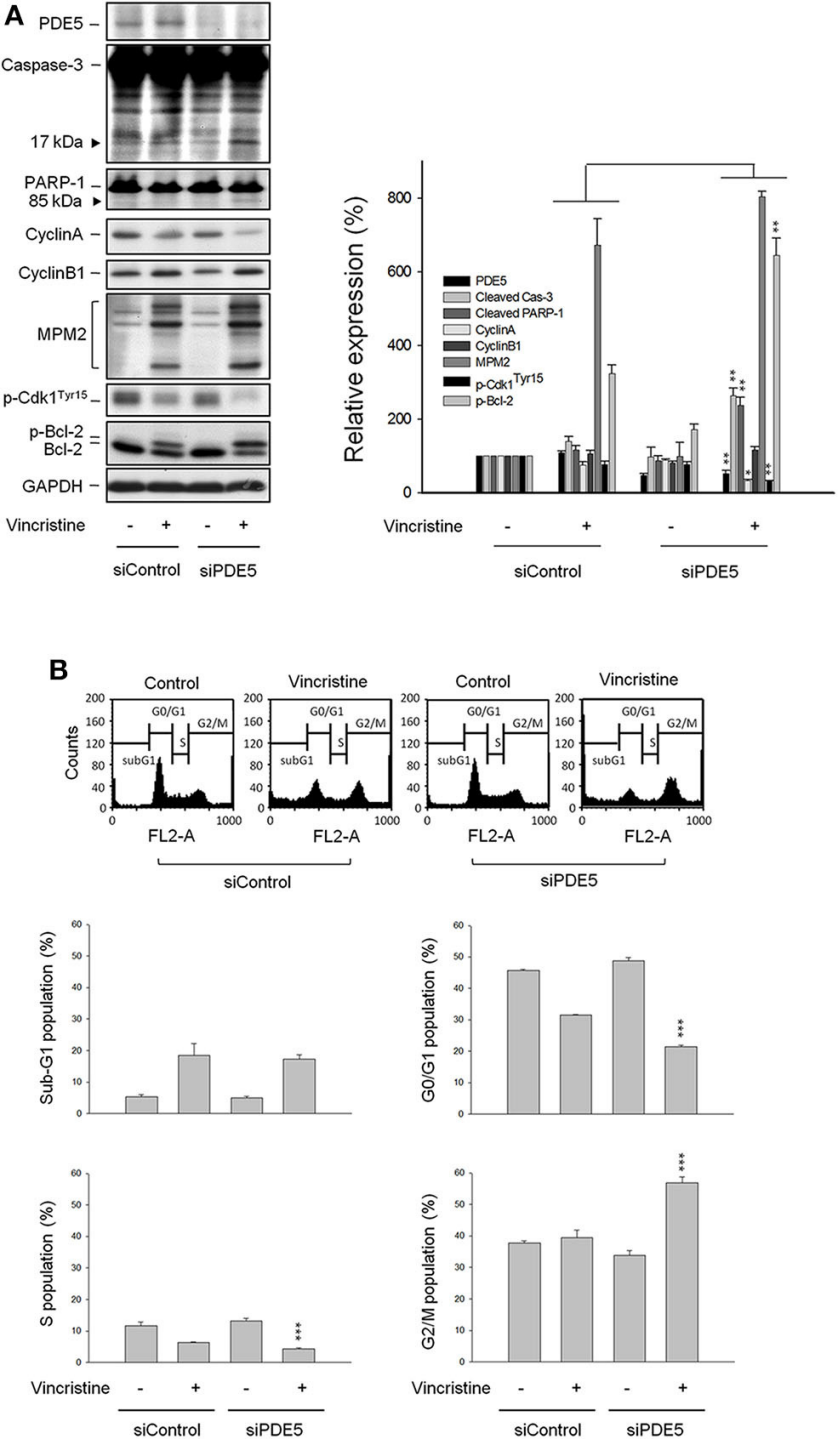
Effect of PDE5 knockdown on vincristine-induced cell-cycle distribution and protein expression. PC-3 cells were transfected with control or PDE5 siRNA as described in the Materials and methods section. After the transfection, the cells were incubated in the absence or presence of vincristine (10 nM) for 24 h and then the cells were harvested and lysed for the detection of protein expression by Western blot analysis **(A)** or the cells were harvested for propidium iodide staining to analyze the distribution of cell populations at various phases using FACScan flow cytometric analysis **(B)**. Data are expressed as mean ± SEM of four independent determinations. ^*^P < 0.05, ^**^P < 0.01, ^***^P < 0.001 compared with vincristine alone at siControl group.

Because the PDE5 inhibitors used in this study (i.e., sildenafil, vardenafil, and tadalafil) displayed similar sensitization activity to vincristine action, the *PDE5A* gene knockdown by siRNA in PC-3 cells was performed to realize its functional role. The data showed an efficient knockdown of PDE5A gene and, therefore, a dramatic reduction of PDE5A protein expression was observed (Figure 4A). The inhibition of PDE5A protein expression significantly amplified several cellular signals stimulated by vincristine, including caspase-3 activation, PARP-1 cleavage, downregulation of cyclin A protein expression, decreased phosphorylation of Cdk1^Tyr15^, and increased Bcl-2 phosphorylation. The upregulation of cyclin B1 and an increase of mitotic-specific MPM-2 phosphorylation, although not significantly, also were observed in PDE5A knockdown cells (Figure 4A). Vincristine-induced mitotic arrest of the cell cycle was markedly increased in PDE5A knockdown cells although sub-G1 population was not augmented (Figure 4B). Altogether, the data indicated that knockdown of *PDE5A* played a crucial role on sensitizing vincristine-induced mitotic arrest and subsequent signaling pathway. However, it was noteworthy that none of the conditions, including vincristine alone, sildenafil alone, or their combination, significantly induced an increase of intracellular cGMP levels in PC-3 cells. In contrast, the positive control (the phosphodiesterase inhibitor 3-isobutyl-1-methylxanthine plus the nitric oxide donor sodium nitroprusside) produced a 23-fold increase of intracellular cGMP (data not shown). The data questioned the functional role of cGMP.

### Sildenafil Potentiates Vincristine-Induced Phosphorylation and Cleavage of BUBR1 and Loss of Tension Across the Sister Kinetochores

**Figure 5:**
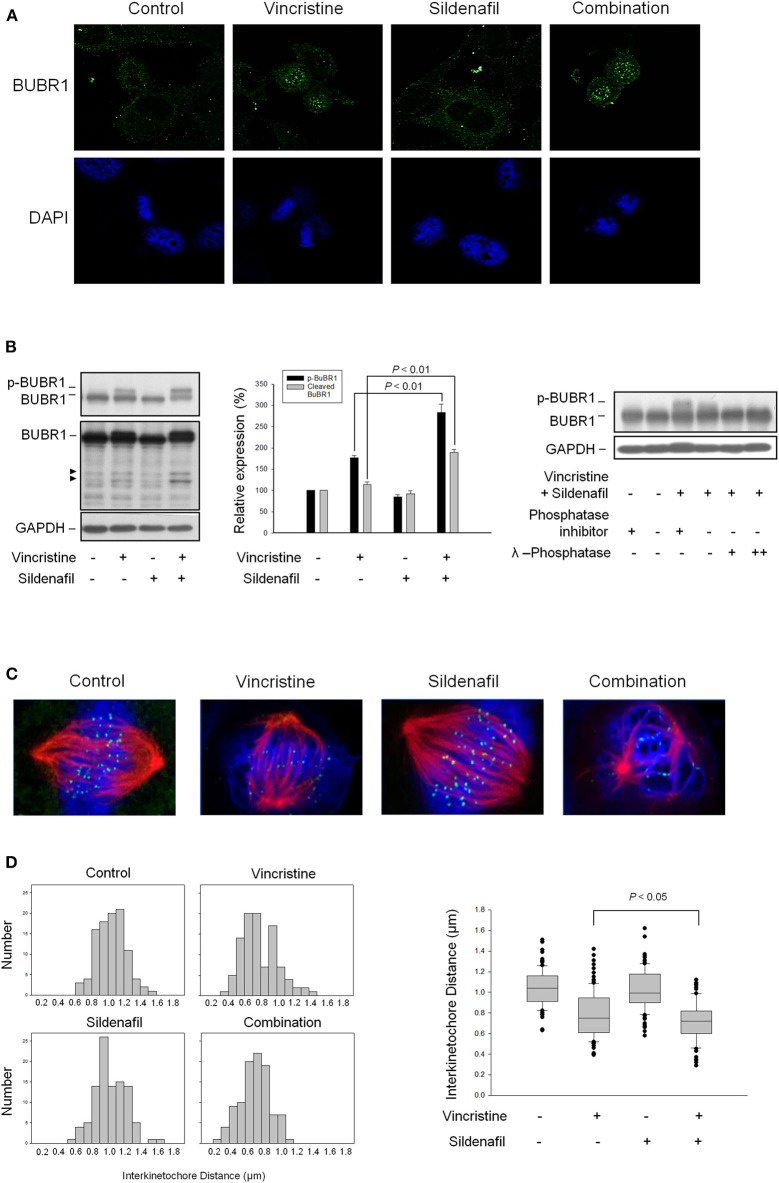
Effect of sildenafil and vincristine on BUBR1 expression and the distance between kinetochore pairs in PC-3 cells. The cells were incubated in the absence or presence of sildenafil (10 μM) and/or vincristine (10 nM) for 24 h. The confocal immunofluorescence examination was performed to detect BUBR1 (green) and chromosome (blue) using BUBR1 antibody and DAPI, respectively **(A)**, or the cells were harvested and lysed for the detection of protein expression by Western blot analysis **(B)**. Data are expressed as mean ± SEM of three independent determinations. Furthermore, BUBR1 phosphorylation was validated by the absence of phosphatase inhibitors or the presence of phosphatase. Both conditions significantly decreased the phosphorylation levels. **(C)** Confocal immunofluorescence microscopic examination was performed to detect kinetochore (green), microtubule (red), and chromosome (blue) using CENP-A antibody, β-tubulin antibody, and DAPI, respectively. **(D)** The distances between paired kinetochores (*n* = 100) were blindly measured at individual z planes, scored from 5 to 8 cells.

BUBR1, a multidomain protein kinase involving in SAC function and chromosome segregation (26), localizes to kinetochore and plays a crucial role in inhibiting anaphase-promoting complex/cyclosome (APC/C), delaying the anaphase onset in guaranteeing accurate chromosome segregation. BUBR1 is expressed with a high mitotic index and its phosphorylation is regulated during mitotic checkpoint activation (28). The images in Figure 5A show that vincristine and in combination with sildenafil induced profound BUBR1 expression. Besides, the phosphorylation of BUBR1 was induced by vincristine and was markedly amplified in the presence of sildenafil. The cleavage of BUBR1 was significantly evoked as well (Figure 5B). Furthermore, BUBR1 phosphorylation was validated by the absence of phosphatase inhibitors or the presence of phosphatase. Both conditions almost completely abolished the phosphorylation of BUBR1 (Figure 5B).

The kinetochore provides signaling function to modify the properties of spindle checkpoint and evokes signal transduction leading to the blockade of anaphase-promoting complex and cell-cycle arrest (29). The images in Figure 5C show that all attached kinetochores on the chromosomes were properly aligned at metaphase plate in control cells, whereas vincristine alone and vincristine combined with sildenafil caused misalignment of chromosomes and attached kinetochores (Figure 5C). Because the tension generated between paired kinetochores was suggested to be proportional to their distance, the distance between sister kinetochore pairs was examined accordingly. The data demonstrated 1.039 ± 0.018 μm in control group. In contrast, the distance was decreased to 0.784 ± 0.023 μm in vincristine alone group. Vincristine combined with sildenafil further significantly reduced the distance to 0.720 ± 0.018 μm (Figure 5D). The data indicated that sildenafil exacerbated vincristine-induced perturbation of microtubule–kinetochore interactions.

### Sildenafil Dramatically Potentiates Vincristine in Suppressing Tumor Growth in Mouse Xenograft Models

**Figure 6:**
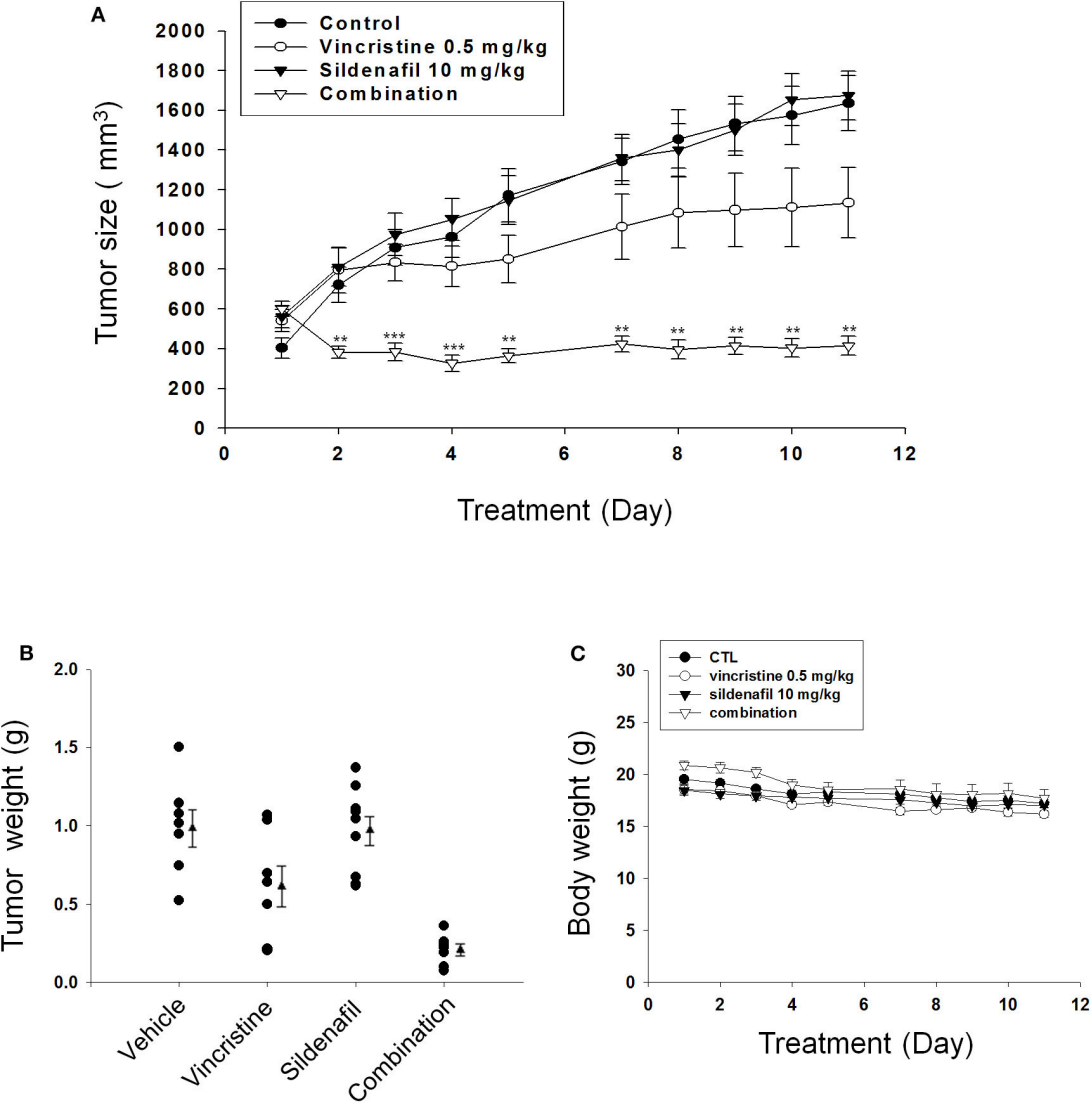
Effect of sildenafil and vincristine on tumor growth in an *in vivo* anti-tumor xenograft model. The nude mice were subcutaneously injected with PC-3 cells (10^7^ cells/mouse). The tumors were measured every day. When the tumors reached to a volume of 400–600 mm^3^, the mice were divided into four groups and the drug administration was initiated as described in the Materials and methods section. **(A)** The length (*l*) and width (*w*) of the tumor were measured, and tumor volume was calculated as *lw*^2^/2. The tumor weights **(B)** and the body weights **(C)** also were measured. Data are expressed as mean ± SEM. ^**^P < 0.01 and ^***^P < 0.001 compared with vincristine alone.

The tumor xenografts in nude mice after subcutaneous PC-3 inoculation were performed. The mice were administered with vehicle, vincristine, sildenafil, or vincristine plus sildenafil when the tumor size reached to an average of about 500 mm^3^ (control group, 403 ± 51; vincristine group, 542 ± 55; sildenafil group, 561 ± 57; combination group, 600 ± 39). Vincristine alone and combined with sildenafil inhibited tumor growth with T/C (treatment/control) ratios of 0.69 and 0.25, respectively, at end-of-treatment (Figure 6A). The average tumor weights at end-of-treatment were 994.7 ± 116.8, 623.5 ± 132.2, 969.9 ± 92.2, and 207.6 ± 36.7 mg in control group, vincristine group, sildenafil group, and combination group, respectively. The median tumor weights were 1101.7, 641.6, 1046.5, and 225.1 mg, respectively (Figure 6B). The data suggested that sildenafil synergized with vincristine on suppressing tumor growth in an *in vivo* model. There was a progressive loss of weight in all experimental animal groups; however, no significant between-group difference was detected (Figure 6C).

## Discussion

Microtubule-targeting agents, such as taxanes and *Vinca* alkaloids, are used in treating a wide variety of cancers through disturbing microtubule dynamics, resulting in mitotic arrest and cell death. The data in this study showed that vincristine induced mitotic arrest with abnormal features of mitosis. The mitotic index was provided demonstrating that vincristine caused predominantly an increase of abnormality in metaphase of the cell cycle. Vincristine induced several cellular events that are crucial during mitotic arrest, including the activation of Cdk1, upregulation of cyclin B1, and phosphorylation of MPM-2 and stathmin. Furthermore, vincristine induced the alteration of several Bcl-2 family members, the loss of mitochondrial membrane potential, and activation of caspase cascades. Altogether, the data suggested that vincristine induced the mitotic arrest of the cell cycle and apoptotic cell death. However, accumulating evidence shows that vincristine treatment is limited by its side effects, in particular several forms of neuropathy. Combination therapy is an efficient therapeutic approach to achieve drug efficacy through lower doses that produce lower toxicity. Sildenafil has been suggested to improve nerve function and to ameliorate long-term peripheral neuropathy (30, 31). Because of its beneficial role, the study aims to repurpose sildenafil as a supportive anticancer agent when combined with vincristine to sensitize tumor killing efficacy. The data demonstrated that sildenafil dramatically increased the mitotic index to vincristine action. All the cellular signals during SAC activation and mitochondrial damage in response to vincristine were synergistically amplified. Notably, other PDE5 inhibitors, such as vardenafil and tadalafil, mimicked sildenafil on potentiating vincristine-induced mitotic arrest and caspase-dependent apoptosis. The data together with PDE5A gene knockdown study supported that the inhibition of PDE5A played a crucial role on sensitizing vincristine-induced mitotic arrest and subsequent apoptotic signaling pathway. However, either sildenafil alone or vincristine plus sildenafil did not induce an increase of intracellular cGMP levels. The data questioned the functional role of cGMP. Because it is reported that sildenafil sensitivity of PDE5 can be regulated by cGMP-independent mechanisms (32), the role of PDE5 and its dependence on cGMP needs further elucidation.

Not only mitotic cell death, vincristine has been suggested to induce cytotoxicity by interfering with interphase microtubules, such as G1 phase (33). It remained unclear whether G1 interphase cytotoxicity was related to vincristine-induced neuropathy. However, our data by double thymidine block and cell synchronization assay revealed that sildenafil significantly reduced G1 cell population in response to vincristine, and increased cells in mitotic arrest and apoptotic death. The data also provided a rationale for the consideration of combination therapy. Nevertheless, it is noteworthy that cells may die in mitosis or exit mitosis as mitotic slippage. Two cellular networks are key players to dictate the cell fate either to death in mitosis or undergoing mitotic slippage: one involving caspase activation and the other is protecting cyclin B1 from degradation (33, 34). Gascoigne and Taylor have reported an excellent study showing that slowing down caspase activation leads to the delay of mitotic cell death; during this time, cyclin B1 keeps progressively being degraded that ultimately permits slippage. In contrast, cyclin B1 overexpression prolongs the duration of mitotic arrest that gives more time for accumulating death signals and ensures cell death (34). Our data were consistent with the notion showing that sildenafil profoundly amplified vincristine-induced cyclin B1 upregulation, which mediated Bcl-2 phosphorylation as a functional link to mitochondrial damage and caspase-dependent cell death. The data validated that sildenafil delayed the mitotic slippage during vincristine exposure and guaranteed longer mitotic arrest and more cell death.

SAC supervises microtubule and kinetochore interactions during the transition of metaphase to anaphase, working on keeping genome stability through delaying cell division only when precise chromosome segregation can be ensured. Mitotic checkpoint complex, the main effector of SAC, is composed of Bub3 (Budding Uninhibited by Benzimidazole 3), BubR1, Mad2 (Mitotic arrest deficient), and CDC20 (Cell division cycle 20). The mitotic checkpoint complex inhibits APC/C activity (an E3 ubiquitin ligase) and prevents proteolytic degradation of securin (an inhibitor of separase) and cyclin B1 (a Cdk1 activator), resulting in the inhibition of separase activity and sustained Cdk1 activity (26, 35, 36). Because cohesin cleavage by separase is required for anaphase and cytokinesis, the mitotic checkpoint complex acts to prevent cohesin cleavage and sister chromatid separation. SAC is induced in the presence of unattached kinetochores and/or a lack of tension between sister kinetochores (26, 36, 37). Our data showed that vincristine reduced the distance between sister kinetochore pairs, indicating the perturbation of microtubule–kinetochore interactions and SAC activation. Notably, sildenafil markedly exacerbated vincristine-induced effects, reinforcing the mitotic arrest at metaphase and subsequent cell death.

BubR1 phosphorylation is critical for checkpoint inhibition of APC/C. During SAC, BubR1 phosphorylation by several kinases including Cdk1, polo-like kinase (Plk1), Aurora B, and monopolar spindle 1 (Mps1) is necessary to supervise the microtubule–kinetochore binding and to detect kinetochore tension, suggesting the key role on kinetochore attachments and checkpoint regulation (38). Moreover, BubR1 has been implicated in drug resistance. Kita et al. reported that BubR1 knockdown in Hela cells showed reduced formation of mitotic checkpoint complex and mitotic arrest induced by thio-dimethylarsinic acid. The mitotic index was significantly decreased associated with almost completely abolished cyclin B1 protein expression in the BubR1 knockdown cells, leading to an increased cell survival when exposed to thio-dimethylarsinic acid (39). Our data were consistent with this notion showing that sildenafil significantly amplified vincristine-mediated BubR1 phosphorylation and mitotic index, increasing cyclin B1 protein levels and ultimately sensitizing cell apoptosis. Furthermore, Kim et al. reported that the inhibition of caspase activity blocked BubR1 cleavage and prolonged mitosis. They showed that the mutation of caspase cleavage sites in BubR1 which prevented BubR1 from the cleavage led to increased aneuploidy and also reduced the rate of cell death when exposed to nocodazole (40). Our data showed that the cleavage of BubR1 was apparent, in particular in cells exposed to vincristine plus sildenafil that also triggered massive caspase activation. The data supported the caspase activation as a determinant of BubR1 cleavage.

It was noteworthy that our supplementary data showed that sildenafil did not synergize both paclitaxel- and docetaxel-mediated effect (Supplementary Figure 4). Precise chromosome segregation is dependent on the SAC. Aurora B plays a key role in the SAC to trigger rapid kinetochore localization of Mps1, granting Mps1 to generate the SAC signals. Anti-mitotics work through disturbing the spindle assembly that induces the SAC and mitotic arrest. However, it is not clear whether there is discrepancy in SAC signals between the stresses of microtubule stabilizing agents and polymerization inhibitors. Gurden et al. have reported that Mps1 inhibition can rapidly override both a nocodazole- and paclitaxel-induced arrest, whereas Aurora B inhibition can only override a paclitaxel-induced arrest through the detection of mitotic index and formation of mitotic checkpoint complex (41). Furthermore, it has been reported that weakened spindle checkpoint with decreased BUBR1 expression is associated with acquired paclitaxel resistance in ovarian carcinoma cells (42). Currently, our study has not yet explained why sildenafil does not synergize both paclitaxel- and docetaxel-mediated effect. However, there exists a discrepancy with the SAC signaling in cells exposed to different anti-mitotics. The sildenafil-mediated different regulation on anti-mitotic sensitivity in this study needs further investigation.

Finally, nude mice xenograft model was used to determine the *in vivo* anti-tumor efficacy. The present work showed that the administration of vincristine combined with sildenafil dramatically inhibited the tumor growth with a low T/C of 0.25 and about 80% inhibition of tumor growth by detecting both average and median tumor sizes.

## Conclusions

The data suggest that sildenafil, in a PDE5-dependent manner, potentiates vincristine-induced mitotic arrest signaling, and sensitizes mitochondria damage–involved apoptosis in CRPC. Both *in vitro* and *in vivo* data suggest the combination potential of PDE5 inhibitors and vincristine on CRPC treatment.

## Data Availability Statement

All datasets generated for this study are included in the article/Supplementary Material.

## Ethics Statement

The animal study was reviewed and approved by The Animal Care and Use Committee at National Taiwan University. All animal procedures and protocols were approved by AAALAC-accredited facility.

## Author Contributions

S-PL and J-HG contributed to the conception and design of the experiments. J-LH performed the experiments and analyzed the data. W-JL, L-CH, and C-HH participated in the progress reports and troubleshooting in experiments. J-HG wrote the article. All authors contributed to the article and approved the submitted version.

## Conflict of Interest

The authors declare that the research was conducted in the absence of any commercial or financial relationships that could be construed as a potential conflict of interest.
