# Supplementary figures and images for: Phosphodiesterase Type 5 Inhibitors Synergize Vincristine in Killing Castration-Resistant Prostate Cancer Through Amplifying Mitotic Arrest Signaling
Source: Front Oncol. 2020 Aug 7;10:1274. doi: 10.3389/fonc.2020.01274 (PMC7427565; doi:10.3389/fonc.2020.01274)

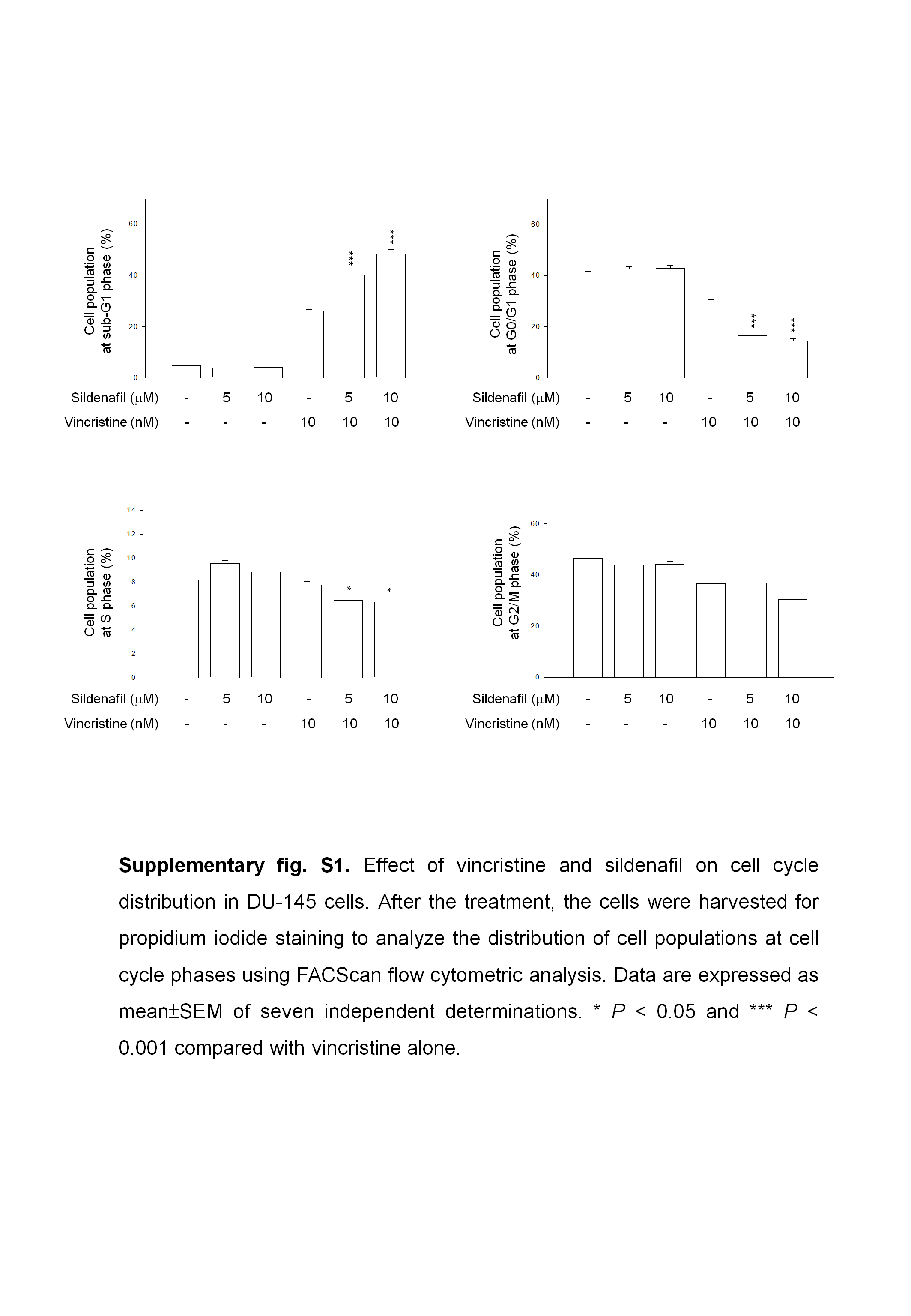

Supplement: Supplementary file 1 [file Image_1.TIF]

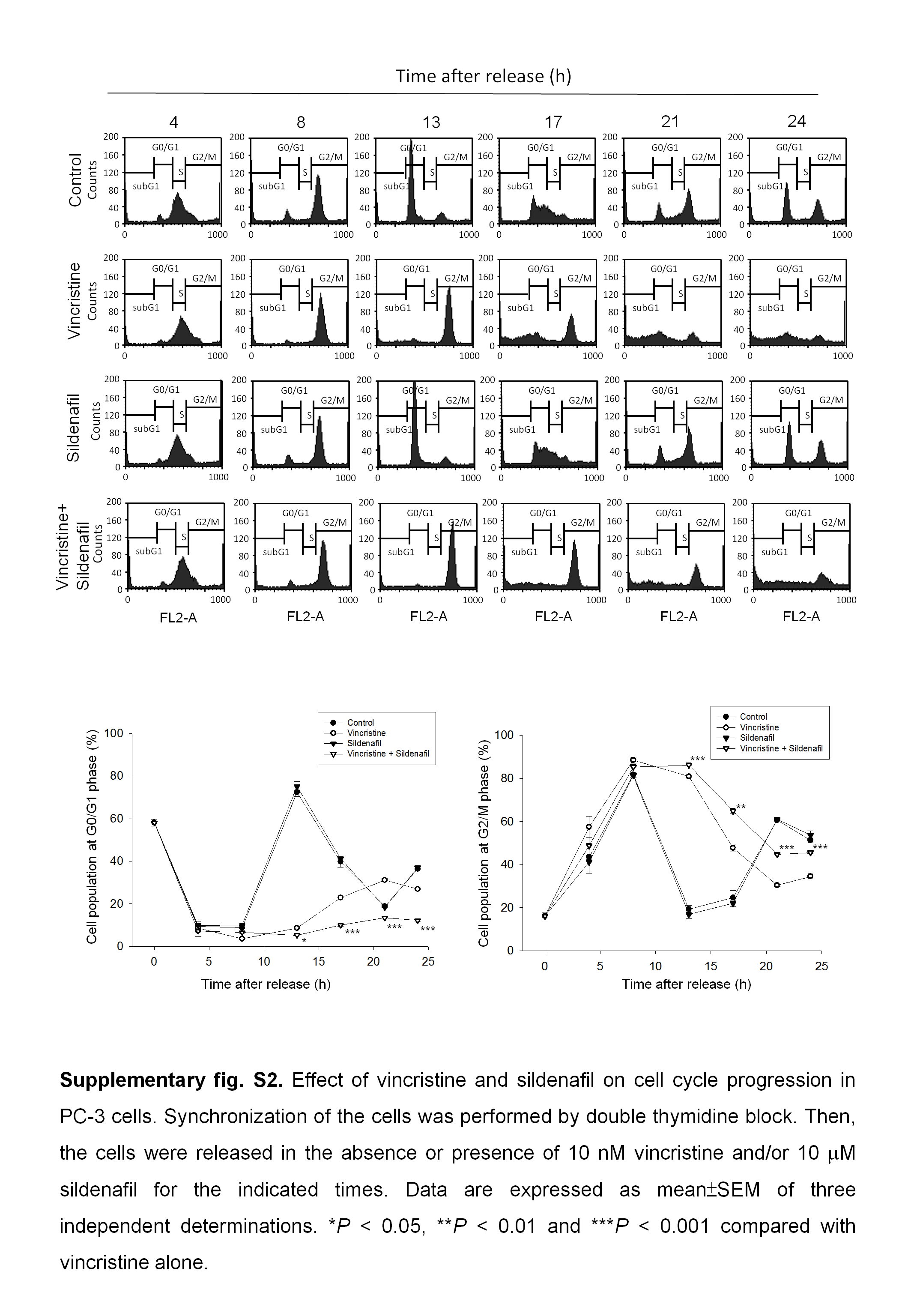

Supplement: Supplementary file 2 [file Image_2.TIF]

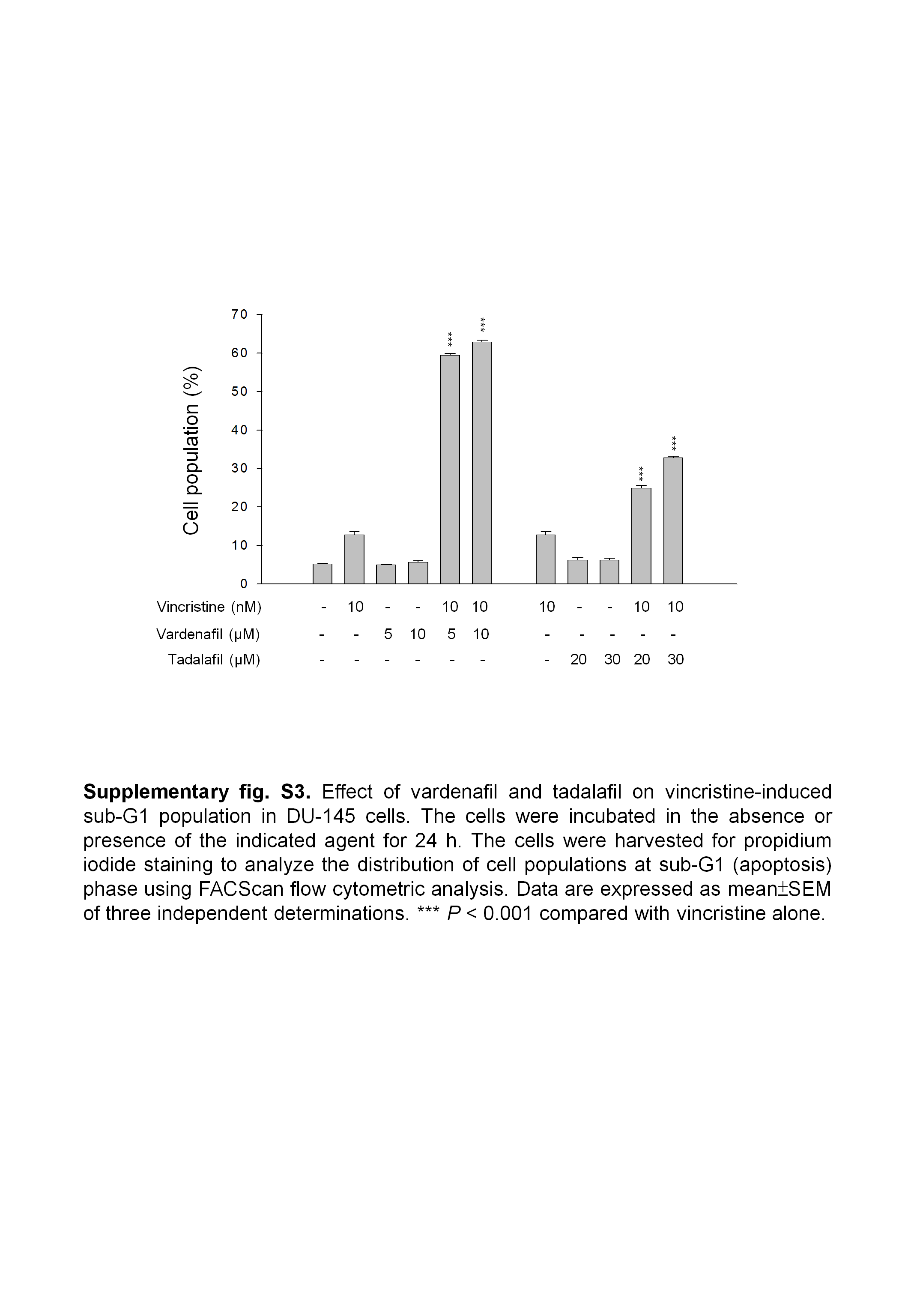

Supplement: Supplementary file 3 [file Image_3.TIF]

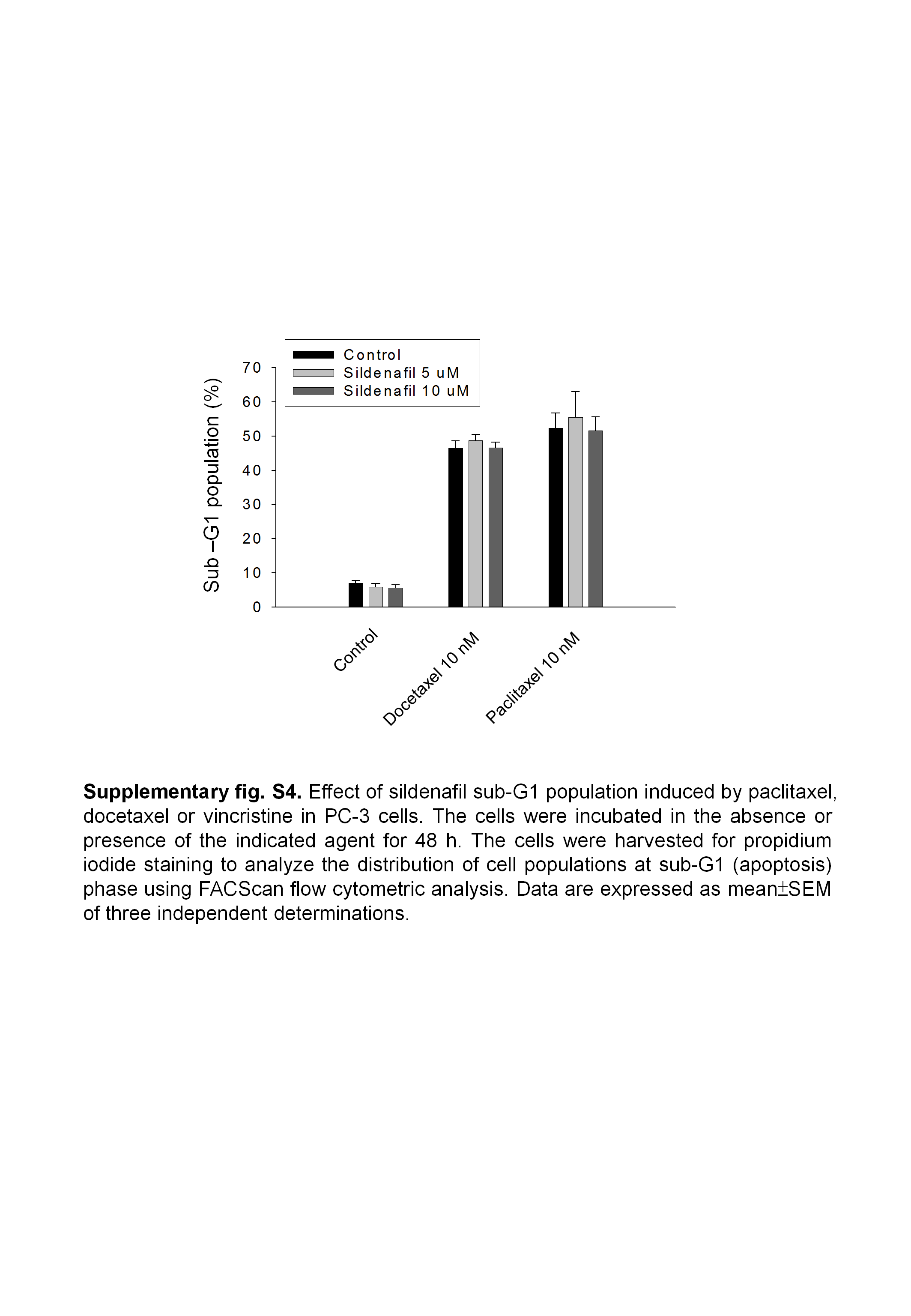

Supplement: Supplementary file 4 [file Image_4.TIF]
